# Differential Antifungal Activity of Human and Cryptococcal Melanins with Structural Discrepancies

**DOI:** 10.3389/fmicb.2017.01292

**Published:** 2017-07-11

**Authors:** Néstor Correa, Cristian Covarrubias, Paula I. Rodas, Germán Hermosilla, Verónica R. Olate, Cristián Valdés, Wieland Meyer, Fabien Magne, Cecilia V. Tapia

**Affiliations:** ^1^Programa de Microbiología y Micología, Instituto de Ciencias Biomédicas, Facultad de Medicina, Universidad de Chile Santiago, Chile; ^2^Facultad de Medicina Clínica Alemana, Universidad del Desarrollo Santiago, Chile; ^3^Escuela de Tecnología Médica, Universidad San Sebastián Santiago, Chile; ^4^Laboratorio de Nanomateriales, Facultad de Odontología, Universidad de Chile Santiago, Chile; ^5^Center for Integrative Medicine and Innovative Science, Facultad de Medicina, Universidad Andres Bello Santiago, Chile; ^6^Instituto de Química de Recursos Naturales, Universidad de Talca Talca, Chile; ^7^Center for Systems Biotechnology, Fraunhofer Chile Research Santiago, Chile; ^8^Laboratorio de Fisicoquímica, Universidad de Talca Talca, Chile; ^9^Molecular Mycology Research Laboratory, Centre for Infectious Diseases and Microbiology, Marie Bashir Institute for Infectious Diseases and Biosecurity, Sydney Medical School – Westmead Hospital, Westmead Institute for Medical Research, University of Sydney, Sydney NSW, Australia; ^10^Laboratorio de Clínica Dávila Santiago, Chile

**Keywords:** melanin, antifungal activity, cryptococcosis, *Cryptococcus* spp., physical-chemical

## Abstract

Melanin is a pigment found in all biological kingdoms, and plays a key role in protection against ultraviolet radiation, oxidizing agents, and ionizing radiation damage. Melanin exerts an antimicrobial activity against bacteria, fungi, and parasites. We demonstrated an antifungal activity of synthetic and human melanin against *Candida* sp. The members of the *Cryptococcus neoformans and C. gattii* species complexes are capsulated yeasts, which cause cryptococcosis. For both species melanin is an important virulence factor. To evaluate if cryptococcal and human melanins have antifungal activity against *Cryptococcus* species they both were assayed for their antifungal properties and physico-chemical characters. Melanin extracts from human hair and different strains of *C. neoformans* (*n* = 4) and *C. gattii* (*n* = 4) were investigated. The following minimum inhibitory concentrations were found for different melanins against *C. neoformans* and *C. gattii* were (average/range): 13.7/(7.8–15.6) and 19.5/(15.6–31.2) μg/mL, respectively, for human melanin; 273.4/(125–>500) and 367.2/(125.5–>500) μg/mL for *C. neoformans* melanin and 125/(62.5–250) and 156.2/(62–250) μg/mL for *C. gattii* melanin. Using Scanning Electron Microscopy we observed that human melanin showed a compact conformation and cryptococcal melanins exposed an amorphous conformation. Infrared spectroscopy (FTIR) showed some differences in the signals related to C-C bonds of the aromatic ring of the melanin monomers. High Performance Liquid Chromatography established differences in the chromatograms of fungal melanins extracts in comparison with human and synthetic melanin, particularly in the retention time of the main compound of fungal melanin extracts and also in the presence of minor unknown compounds. On the other hand, MALDI-TOF-MS analysis showed slight differences in the spectra, specifically the presence of a minor intensity ion in synthetic and human melanin, as well as in some fungal melanin extracts. We conclude that human melanin is more active than the two fungal melanins against Cryptococcus. Although some physico-chemical differences were found, they do not explain the differences in the antifungal activity against *Cryptococcus* of human and cryptococcal melanins. More detailed studies on the structure should be considered to associate structure and antifungal activity.

## Introduction

**FIGURE 1:**
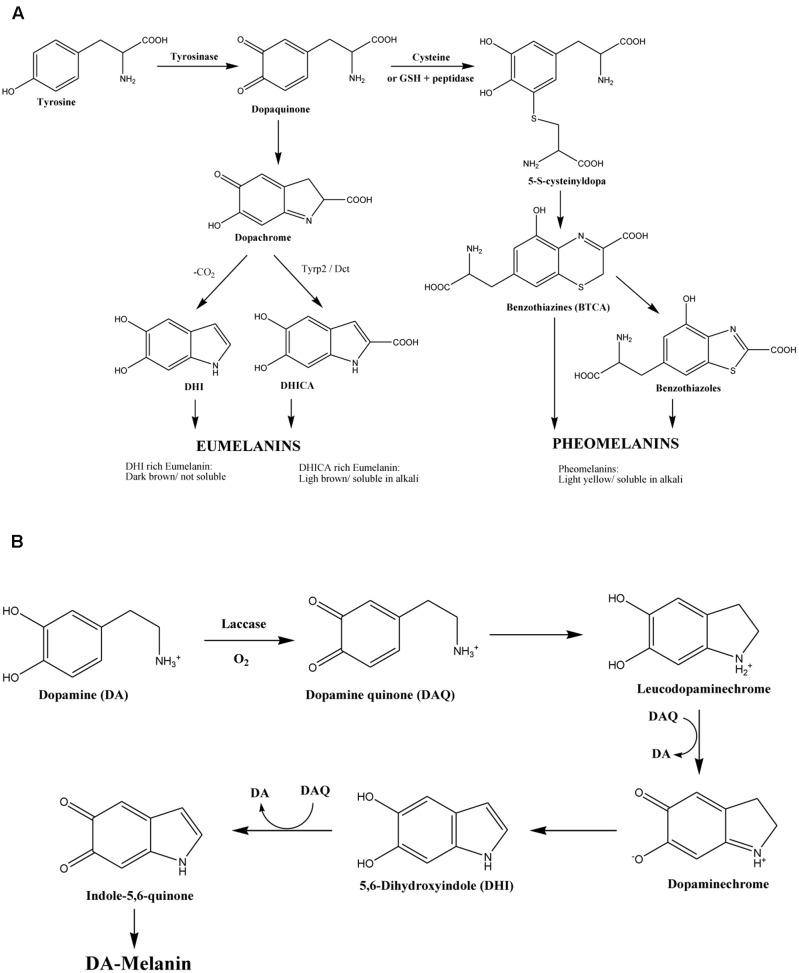
**(A)** Biosynthetic pathway of human melanin (Alaluf et al., 2002) and **(B)** of *Cryptococcus neoformans* melanin (Williamson et al., 1998).

Melanin is a pigment that can be found in all biological kingdoms, largely recognized for play an important role in the protection against ultraviolet light (UV), oxidizing agents and ionizing radiation (Eisenman and Casadevall, 2012). It also participates in other important functions, including thermoregulation in lower vertebrates, camouflage, and sexual attraction in some species (Ito and Wakamatsu, 2003). In human beings this pigment is made up of three different components: pheomelanin, of light yellow or red color, soluble in alkali; eumelanin rich in 5.6-dihidroxiindol-2-carboxylic acid (DHICA), of light brown color, soluble in alkali and eumelanin rich in 5,6-dihydroxiindole (DHI) of dark brown or black color. Thus, the wide range of skin tones and human hair are therefore the result of various combinations of the impact of those three factors (Alaluf et al., 2001). Preliminary studies in our laboratory demonstrated that commercial synthetic melanin (Sigma–Aldrich^TM^) had an antifungal activity against *Candida albicans* and other *Candida* species, such as *C. parapsilosis* strain 22019, *C. glabrata* strain 2001, *C. krusei* strain 6258 (Fuentes et al., 2014). Interestingly, an inhibiting activity has also observed in isolates of *Candida* spp. which are resistant to fluconazol (Fuentes et al., 2014). Nevertheless, the physicochemical structure of melanin is still scarcely known (Wang et al., 1995; Williamson et al., 1998; Casadevall et al., 2000; Kumar et al., 2011; Eisenman and Casadevall, 2012; Banerjee et al., 2014; Nosanchuk et al., 2015) since it is a highly insoluble compound in aqueous and organic solvents, and moreover its solubilisation modifies its structure (Nosanchuk et al., 2015). Compared to fungal melanin, the human melanin synthesis uses distinct intermediate products and enzymes (**Figures 1A,B**), which suggests that melanin may possess a different structure; resulting in various activities to the organisms it originated accordingly.

Cryptococcosis is a systemic infection acquired by the inhalation of the encapsulated yeasts *Cryptococcus neoformans* or *C. gattii* or less frequently by its ingestion or inoculation of the skin (Gullo et al., 2013). The cosmopolitan distribution and habitats of *Cryptococcus* spp. include bird droppings, especially from pigeons for *C. neoformans*, favored by the presence of high concentration of nitrogen, creatine, and salts. *Cryptococcus gattii* presence is related to decaying wood, such as that found in the bark of *Eucalyptus* spp. (Negroni, 2012; Gullo et al., 2013). The clinical manifestations range from asymptomatic pulmonary colonization to systemic dissemination. The cryptococcal meningitis is caused by *C. neoformans* and is the most severe clinical manifestation and affects mainly patients with acquired immunodeficiency syndrome (AIDS) mainly (Negroni, 2012; Gullo et al., 2013). On the other hand, *C. gattii* infects immunocompetent individuals in tropical and subtropical areas, although cases have been reported in temperate zones (Gullo et al., 2013). The pathogenicity of *Cryptococcus* species has been attributed in part to the synthesis of melanin (Wang et al., 1995).

In *C. neoformans*, the products of the melanization genes are considered as virulence factors, because their contribution to the fungal dissemination from the lungs to other organs (Eisenman and Casadevall, 2012). In the most aggressive *C. gattii* strains, the genes related to the melanin synthesis are overexpressed, and a larger amount of melanin is synthetized (e.g., *LAC1* and *LAC2*) (Eisenman and Casadevall, 2012). Both *C. neoformans* and *C. gattii* synthesize melanin from L-dopa (**Figure 1B**), through an absolute dependence on an exogenous substrate. The genome of both species codes for the laccase enzymes *LAC1* and *LAC2* (Ngamskulrungroj et al., 2011; Chaskes et al., 2014), being *LAC1* the main gene associated with the production of melanin (Williamson et al., 1998). L-dopa is one of the substrates identified in *C. neoformans*, being able to use other catecholamines, such as dopamine, norepinephrine, and D-dopa (Eisenman et al., 2007). In addition, some derivatives from plants such as flavonoids and caffeic acid could be used by both species to produce pigment (Eisenman and Casadevall, 2012).

Although some melanins have a known antifungal activity, in *Cryptococcus* species they are an important virulence factors too. Therefore, the question that arises is: does the cryptococcal melanin have a similar activity than human melanin?. We hypothesized that cryptococcal melanin is less active than human melanin due to their physical-chemical differences. In order to investigate about it, we determined the antifungal activity of human melanin against *Cryptococcus* species compared to synthetic melanin and melanin extracted from the two *Cryptococcus* species. In addition, melanins from human, fungal and synthetic origin were physicochemically characterized by Scanning Electron Microscopy (SEM) and Energy Dispersive X-ray spectroscopy (EDX), Infrared spectroscopy (FTIR), Matrix-Assisted Laser Desorption/Ionization-Time of Flight Mass Spectrometry (MALDI-TOF-MS), and High Performance Liquid Chromatography (HPLC).

## Materials and Methods

### Melanin Extract Preparation

#### Human Melanin

Human melanin was extracted from black hair donated by a volunteer through an acid/base method according to the protocol of Liu et al. (2003). This melanin source was chosen due to its high content of eumelanin (dark melanin). An amount of 14.49 g of human without hair dye and/or colorant, was incubated in 1 M NaOH for 24 h, then it was acidified by adding pure hydrochloric acid and later centrifuged at 1800 ×*g* for 15 min. This process was repeated 10 times. To discard proteins, the extract was boiled for 3 h at acid pH (<2) three times and then it was centrifuged to obtain a clear supernatant. The precipitate was then washed five times with water to reach a neutral pH. Finally, two washes with ethanol and one with alcohol-ether (1:1 [vol/vol]) were carried out to dehydrate the extract. Finally, the extract was dried in an incubator at 65°C for 5 h. A total of 0.532 g of melanin with an efficiency of 3.6% was obtained. This work was approved by the Ethics Board of Clínica Dávila according the Code of Ethics of the World Medical Association (Declaration of Helsinki).

#### Fungal Melanin

Eight strains genetically characterized were used; four *C. neoformans* (VNI WM148, VNII WM626, VNIII WM628, and VNIV WM629) and four *C. gattii* (VGI WM179, VGII WM178, VGIII WM175, and VGIV WM2363) from the culture collection of the Molecular Mycology Research Laboratory at Sydney Medical School, Sydney University were used. All the assays were performed with fungal strains grown on Sabouraud agar (BD^®^) for 24 h at 30°C. To induce the melanization, all *Cryptococcus* strains were grown for 48 h at 30°C on the Niger Seed agar (Shields and Ajello, 1966), where fungal colonies became a dark brown color. Melanin extraction was performed from a PBS (phosphate-buffered saline) suspension of 10^7^ yeasts/mL. Cryptococcal cells were lysed after being hit with glass pearls using a culture beater (Minilys, Bertin Technologies) with seven pulses of 60 s at 3000 rpm. The lysates were centrifuged for 5 min at 10.000 rpm. The cell residues were washed three times with trichloroacetic acid at 5%, twice with alcohol-ether (1:1 [vol/vol]), and once with absolute ether. The residual material was dissolved in 0.05 M of sodium carbonate solution during 10 min incubation at 100°C in a thermo regulated water bath. After centrifugation to remove the insoluble material, the solution was dried at 65°C overnight (Shivprasad and Page, 1989). The dried residue was kept at -20°C before it use. Once the extract was dissolved in a 0.5 M NaOH solution, it was quantified at 400 nm absorbance by using commercial synthetic melanin as a standard (Sigma–Aldrich^TM^).

### Antifungal Activity of Melanin

The antifungal susceptibility testing was performed using a broth microdilution test accordingly to the European Committee for Antimicrobial Susceptibility Testing 7.2 definitive document with modifications (Arikan-Akdagli et al., 2012). For this purpose, 5 mg of all melanin extracts were diluted in 100 μL of 0.5 M NaOH solution to obtain a stock solution. Then, the stock solution was serially diluted in RPMI 2X medium to obtain a final concentration in a range of 0.488 and 500 μg/mL, in a microdilution plate of 96-wells flat bottom. *Cryptococcus* strains were inoculated in the plate at a concentration of 0.5 Mc Farland scale. The minimum inhibitory concentrations (MICs) were determined visually, considering a 50% of inhibition, compared to the growth control (microdilution wells without melanin). The MICs were also checked by plate colonies counting. The antifungal activity of the different melanin extracts was tested against the following strains: the standard strains of *C. neoformans* VNI WM148, VNII WM626, VNIII WM628, and VNIV WM629 and *C. gattii* VGI WM179, VGII WM178, VGIII WM175, and VGIV WM2363. In addition, eight clinical *C. neoformans* strains from a local collection isolated from patients with cryptococcal meningitis were tested. The strains *Candida krusei* strain 6258 and *Candida parapsilosis* strain 22019 were used as controls for the antifungal susceptibility test, based in our previous study (Fuentes et al., 2014). The plates were incubated at 30°C and observed during 72 h after incubation.

### Physical-Chemical Characterization of Melanin Extracts

The extracts were characterized by different analyses using synthetic melanin (Sigma–Aldrich^®^) as standard.

#### Scanning Electron Microscopy (SEM) and Energy Dispersive X-ray Spectroscopy (EDX)

The EDX technique allows analyzing the samples qualitatively and quantitatively based on the peak emission of X-rays resulting from the interaction between each element of a compound and the electron beam. Then, the chemical components of the melanin were determined by EDX. Melanin extracts were fixed in glutaraldehyde 2% with 0.1 M sodium cacodylate buffer during 2 h and then washed once in the same solution. To dehydrate the extracts, they were exposed to increasing alcohol concentrations (50°, 70°, 95°, and 100° for 5 min). Samples were dried using a critical point dryer (Autosamdri^®^-815, Series A) for 45 min through carbon dioxide (CO_2_). Once dried, samples were mounted in aluminum sample holders, and then metalized with gold (Denton Vacuum Desk V). Finally, samples were visualized using SEM (JEOL^®^ Model JSM-IT300LV).

#### Fourier Transform Infrared Spectroscopy (FTIR)

This technique allows studying the molecular composition of melanin via the absorption of the infrared zone of the electromagnetic field. For this, 5 mg of powder melanin samples were directly measured with attenuated total reflection (ATR) technique in an Agilent Cary 630 ATR FTIR (Agilent Technologies) spectrometer. Spectra were recorded in the 400–4000 cm^-1^ wavenumber range by using a resolution of 4 cm^-1^ and 150 sample scans. Samples were analyzed in uniplicates.

#### High Performance Liquid Chromatography (HPLC)

In this work, the protocol described by Sun et al. (2016) was used with some modifications. Briefly, melanin extracts were dissolved in a 0.5 M NaOH solution and passed through a 0.2 μm filter. The HPLC analysis was performed with a Perkin Elmer Series 200 HPLC system, using an UV detector at 260 nm and a Kromasil C18 (4.6 mm × 300 mm, 5 μm) column. The mobile phase consisted in methanol:acetic acid (99:1), that was applied in an isocratic elution during 100 min (flow rate: 0.2 mL/min, injection volume: 20 μL, and column temperature: 25°C).

#### Matrix-Assisted Laser Desorption/Ionization-Time of Flight Mass Spectrometry (MALDI-TOF-MS)

Melanin extracts were analyzed in a MALDI-TOF-MS instrument (Bruker Daltonics) in two mass ranges between 380 and 2,000 Da and 2,000 and 10,000 Da, respectively. Melanin filtered solutions were diluted in acetonitrile (1:9) and homogenized in an ultrasound bath (37 KHz) for 5 min. One microliter of each melanin solution was spotted in a MALDI steel plate and air dried. Then, 1 μL of the matrix α-cyano-4-hydroxycinnamic acid (CHCA, 1% acetonitrile:trifluoroacetic acid = 1:1) was added on the spots and air dried. The mass spectra were processed using flexAnalysis 3.3 software (Bruker Daltonics).

### Statistics

The statistical analyses were performed by using the software GraphPad Prism version 5.01 (GraphPad Software, Inc.) using *t*-student test, ANOVA two-way, and Bonferroni post-test for the antifungal susceptibility testing experiments.

## Results

### Antifungal Activity of Melanin

**FIGURE 2:**
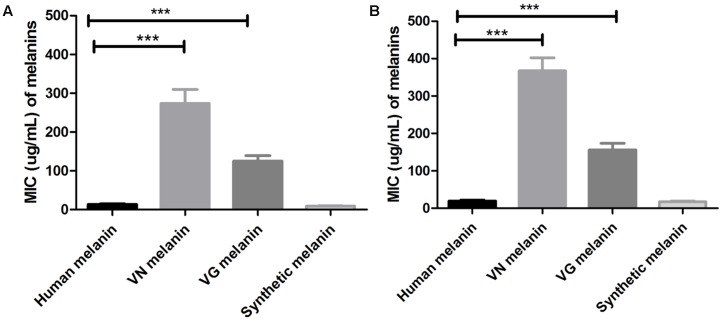
Minimal inhibitory concentrations (MICs) values of different melanins against strains of: **(A)** *C. neoformans* and **(B)** *C. gattii* ^∗∗∗^*p* < 0.0001 (VN, *C. neoformans*; VG, *C. gattii*). *T*-student test, ANOVA two-way test, and Bonferroni post-test. Bars represent mean and standard error of the mean (SEM) of three replicates.

Minimal inhibitory concentration values of the different melanin extracts against *C. neoformans* and *C. gattii* were determined and reported in the **Table 1**. According, to the origin species of the melanin, MIC values (average and range) obtained against *C. neoformans* were: 13.7 (7.8–15.6) μg/mL for human melanin; 273.4 (125 – >500) μg/mL for *C. neoformans* melanin; 125 (62.5–250) μg/mL for *C. gattii* melanin; and 9.2 (3.9–15.6) μg/mL for synthetic melanin (**Figure 2A**). Moreover, MIC values observed against *C. gattii* were: 19.5 (15.6–31.2) μg/mL for human melanin; 367.2 μg/mL (125 – >500) for de *C. neoformans* melanin; 156.2 (62–250) μg/mL for *C. gattii* melanin; and 17.4 (3.9–31.2) μg/mL for synthetic melanin (**Figure 2B**).

**Table 1:** Minimal inhibitory concentrations (MICs) of different melanins againsts *Cryptococcus neoformans* (*n* = 4) and *C. gattii* strains (*n* = 4).

	*C. neoformans*		*C. gattii*	
Source	Range μg/mL	Average μg/mL	Range μg/mL	Average μg/mL
Synthetic melanin	3.9–15.6	9.2	3.9–31.2	17.3
Human melanin	7.8–15.6	13.7	15.6–31.2	19.5
VNI WM148 melanin	250–250	250	>500–>500	>500
VNII WM626 melanin	>500–>500	>500	>500–>500	>500
VNIII WM628 melanin	125–250	156	125–250	218
VNIV WM629 melanin	125–250	187.5	250–250	250
VGI WM179 melanin	125–125	125	125–125	125
VGII WM178 melanin	125–250	187.5	125–250	218.8
VGIII WM175 melanin	62.5–62.5	62.5	62–125	93.6
VGIII WM2363 melanin	62.5–62.5	62.5	62–125	93.6

*The range and average (obtained by triplicate) of MICs for different melanins are shown.*

**FIGURE 3:**
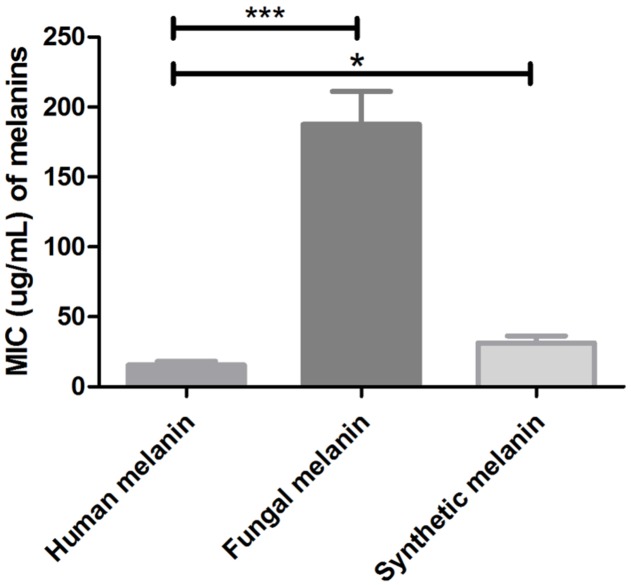
Minimal inhibitory concentrations values of human melanin, fungal melanin (extracted from *C. neoformans* VGIII, strain WN175), and synthetic melanin against clinical strains of *C. neoformans* (^∗^*p* = 0.015; ^∗∗∗^*p* < 0.0001). *T*-student test, ANOVA two-way test, and Bonferroni post-test. Bars represent mean and standard error of the mean (SEM) of three replicates.

Additionally, we tested the antimicrobial activity of the melanin extracts against eight clinical strains of *C. neoformans* and observed MIC values (range) corresponding to 31.4 μg/mL (15.6–62.5) for synthetic melanin; 15.6 μg/mL (7.8–31.2) for human melanin, and 187.5 μg/mL (125–250) for cryptococcal melanin VG III WM175. The human melanin showed a significantly higher antifungal activity against both *C. neoformans* and *C. gattii*, compared to the synthetic and fungal melanins (*p* < 0.0001 and *p* = 0.015) (**Figure 3**).

### Scanning Electron Microscopy (SEM) and Energy Dispersive X-ray Spectroscopy (EDX)

**FIGURE 4:**
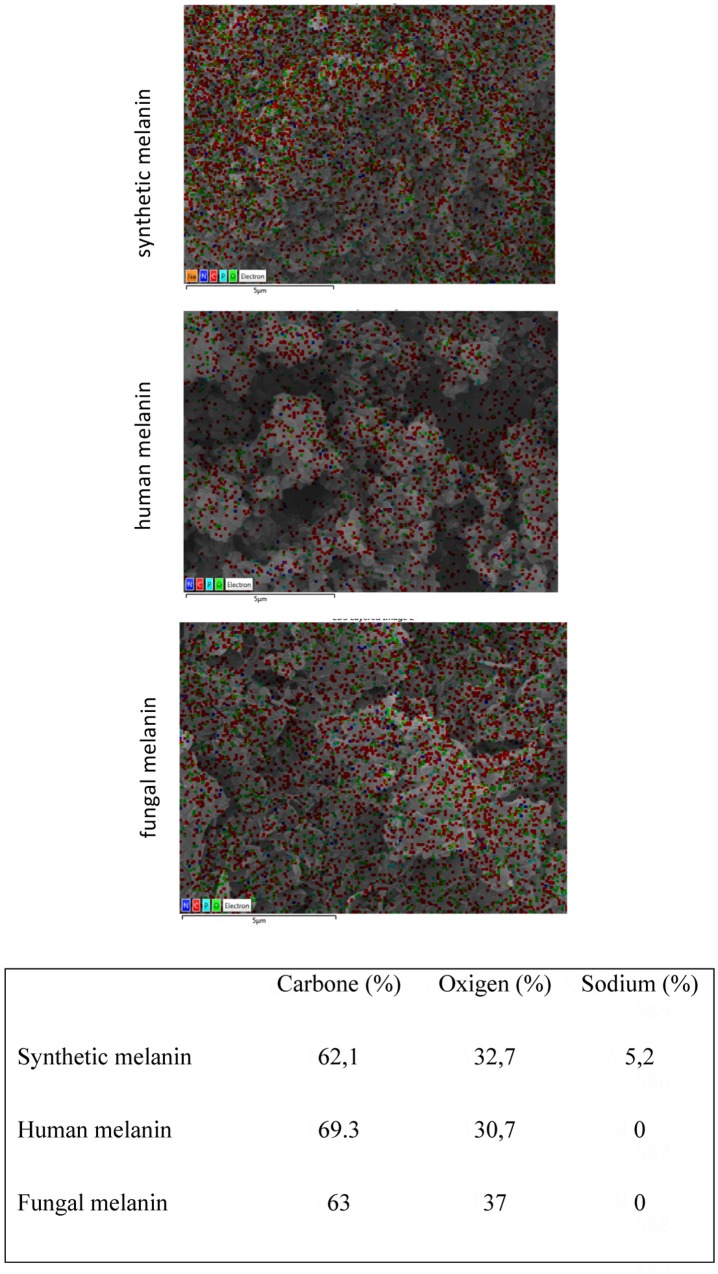
Melanin composition analyzed by Energy Dispersive X-ray spectroscopy (EDX). Red dots: carbon, green dot: oxygen, and blue dots: nitrogen.

For further analyses of melanins from different sources, we used the technique of SEM (data not shown). Our results revealed that the human melanin displays as ellipsoidal deposits in accordance with melanosomal structures, similarly to a previous study (Liu et al., 2003). On the other hand, the structure of the synthetic melanin as well as the melanin obtained from *C. neoformans* (molecular type VNII WM626) shows an amorphous (irregular) shape pattern in comparison with the human melanin. The main elements detected on the surface of melanin were carbon (C) and oxygen (O) which are present at equivalent percentages on each melanin extract analyzed (**Figure 4**). An interesting difference between melanins was the presence of sodium, which was only detected in the synthetic melanin. Melanin particle sizes were estimated from SEM images. Particle size was measured from SEM images (magnification × 33,000) considering an area of 16 μm^2^ and particle number of 30. The mean particle sizes (length × width) for human and fungal melanin were 1 × 0.8 μm (standard deviation [length × width] = 0.074) and for synthetic melanin was 0.6 × 0.3 μm (standard deviation [length × width] = 0.061).

### Fourier Transform Infrared Spectroscopy (FTIR)

**FIGURE 5:**
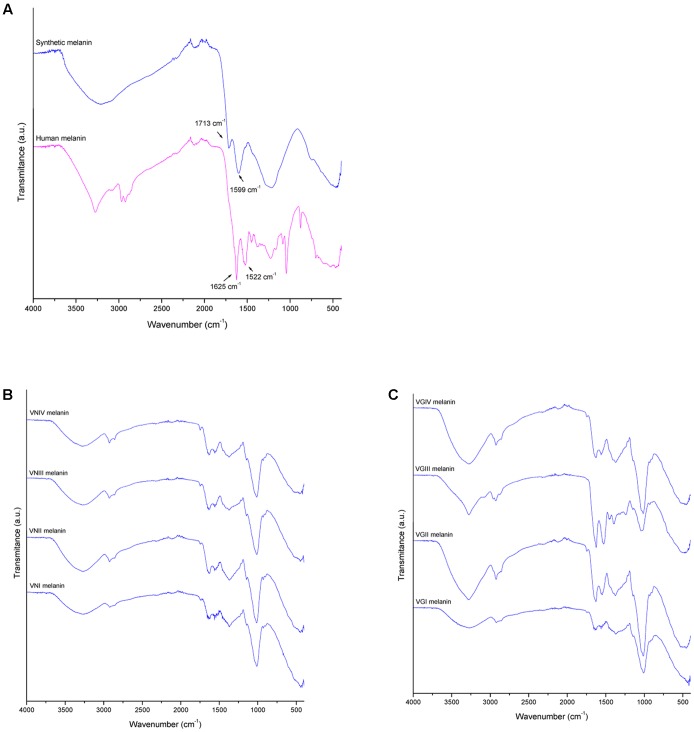
Melanin characterization by fourier transform infrared spectroscopy (FTIR) for **(A)** human melanin (upper blue profile) and synthetic melanin extracts (lower red profile), **(B)** *C. neoformans* melanin extracts (vni, VNI WM148; vnii, VNII WM626; vniii, VNIII WM628; and vniv, VNIV WM 629), and **(C)** *C. gattii* melanin extract (vgi, VGI WM179; vgii, VGII WM178; vgiii, VGIII WM175; and vgiv, VGIV WM2364).

The different melanin extracts were analyzed using FTIR in order to compare their secondary structure. The spectra of melanin obtained from the extracts from different organisms are presented in **Figure 5**. A wide band in zone of 3200 cm^-1^can be observed, corresponding to links vibration of the functional groups -OH and -NH_2_. Other peaks can be observed nearby to 1625 cm^-1^ (C=C, C=N, C=O), 1015 cm^-1^ (C-O) of phenolic or carboxylic groups that characterize the biological origin of melanin. In **Table 2**, the transmission patterns of fungal melanin extracts showed a similar spectroscopic curve, suggesting the presence of similar functional groups. However, differences in the intensity of signals can be observed in the 1625 and 1522 cm^-1^ zone in human and fungal melanins compared to the synthetic one. This is related to C-C links of the aromatic ring of the molecule.

**Table 2:** Fourier transform infrared spectroscopy (FTIR) peaks for different melanins.

Melanin origin	*Peaks* (cm^-1^)^∗^										
Synthetic	3314		3107		1713		1599		1217		
Human		3277		2967		1625	1522			1043	877
VNI WM148 melanin		3265		2920		1628		1369		1010	
VNII WM626 melanin		3279		2923		1628		1369		1013	
VNIII WM628 melanin		3280		2927		1627		1373		1012	
VNIV WM629 melanin		3282		2926		1627		1372		1012	
VGI WM179 melanin		3273		2924		1626		1375		1013	
VGII WM178 melanin		3277		2926		1624	1527	1396		1040	
VGIII WM175 melanin		3280		2923		1628	1545	1375		1012	
VGIV WM2363 melanin		3269		2920		1406		1369	1291	1007	

*^∗^Unique measurement.*

### High Performance Liquid Chromatography (HPLC) and Matrix-Assisted Laser Desorption/Ionization-Time of Flight Mass Spectrometer (MALDI-TOF MS)

**FIGURE 6:**
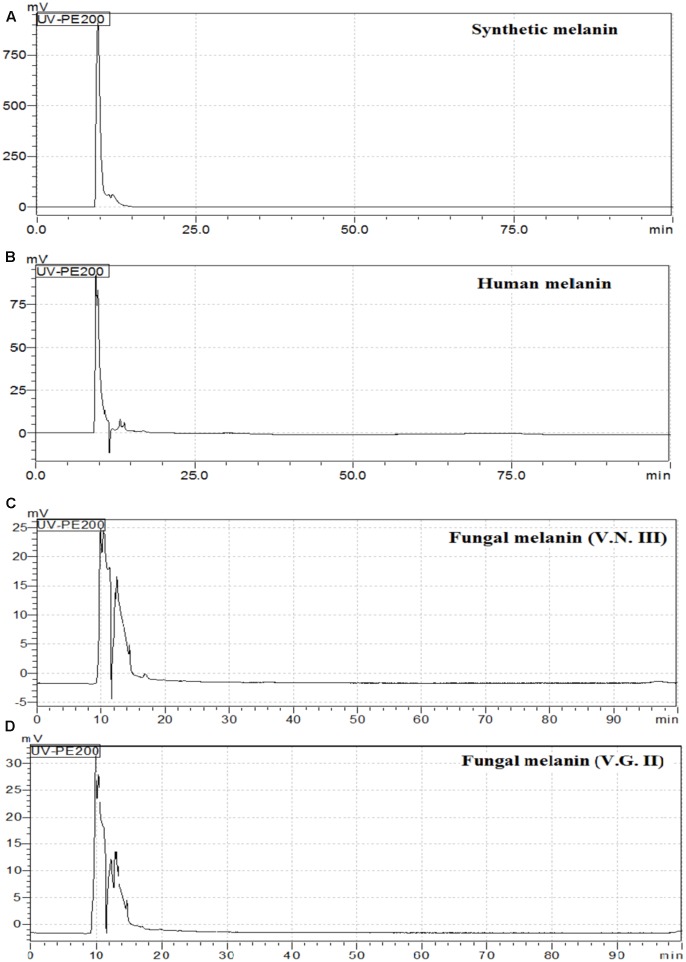
High Performance Liquid Chromatography (HPLC) chromatograms of **(A)** Synthetic melanin, **(B)** human melanin **(C)** fungal melanin extract VN III WM626 and **(D)** fungal melanin extract VG II WM178.

HPLC analysis showed that the chromatograms of synthetic and human melanin are composed by a single and symmetric signal with a retention time of 9.606 and 9.481 min, respectively (**Table 3** and **Figure 6**). The human melanin also exposed a minor peak around 13 min (**Figure 6B**). The chromatograms of fungal melanin were similar to synthetic and human melanin similar to synthetic and human melanin, in terms of the main chromatographic peak, as shown in **Figures 6A,B**. However, all cryptococcal melanin chromatograms exposed another peak (nearby to 14 min) with a variable intensity, suggesting the presence of more than one compound in the fungal extracts (**Figures 6B,C**).

**Table 3:** Retention time of different melanins by High Performance Liquid Chromatography (HPLC).

Melanin	Retention time (min)
Synthetic	9.606
Human	9.481
VNI WM148	9.929
VNII WM626	10.291
VNIII WM628	9.913
VNIV WM629	10.318
VGI WM179	9.799
VGII WM178	9.846
VGIII WM175	10.506
VGIV WM2363	12.013

**FIGURE 7:**
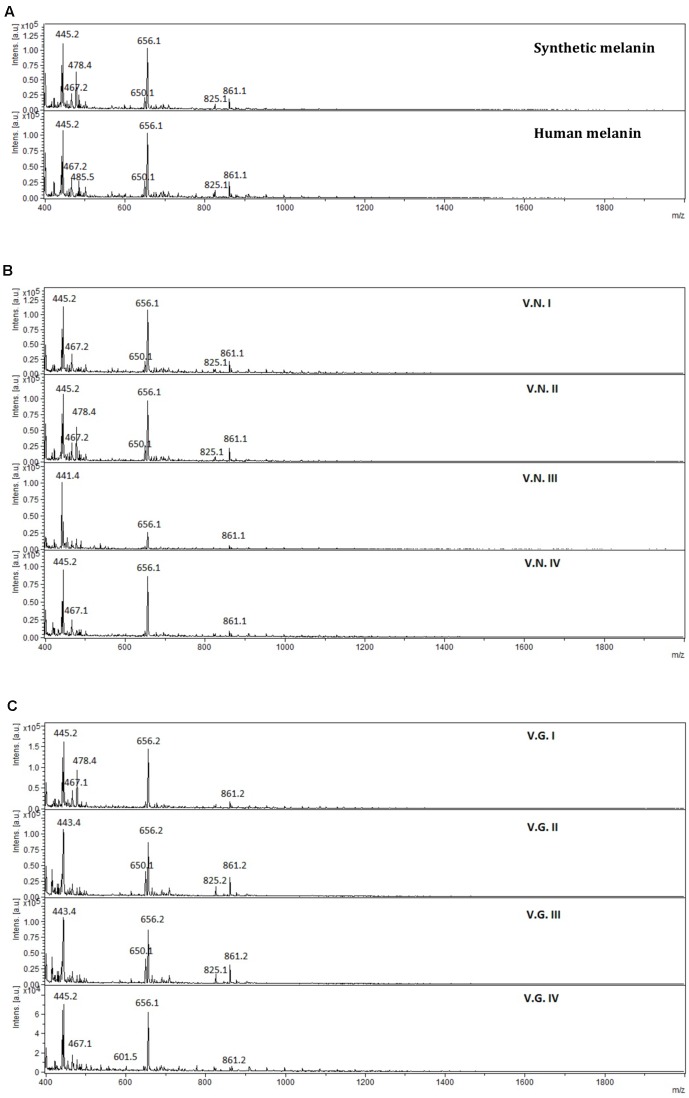
Mass spectra obtained from **(A)** synthetic and human melanin, **(B)** *C. neoformans* (V.N.I, VNI WM148; V.N.II, VNII WM626; V.N.III, VNIII WM628; and V.N.IV, VNIV WM629), and **(C)** *C. gattii* (V.G.I, VGI WM179; V.G.II, VGII WM178; V.G.III, VGIII WM175; and V.G.IV, VGIV WM2363), in the range of 380–2,000 Da.

The mass spectra of melanin extracts obtained by MALDI-TOF-MS analysis (**Figure 7**) revealed that melanin samples had a similar mass pattern in the range between 380 and 2,000 Dalton (Da). It was observed an abundant ion at 656 Da, with high relative intensity in all extracts and also, a less intense ion at 861 Da. Additionally, the mass profiles of synthetic and human melanin, as well as fungal melanins extracts VNI WM178, VNII WM 626, VGII WM178, and VGIII WM175 showed an ion of lower intensity at 825 Da, which was not observed in the rest of the fungal melanins VNIII, WM 628 VNIV, WM629 VGI WM179 and VGIV WM2363. The mass spectrum of the fungal extracts suggested the presence of two or three different compounds, as well as in the HPLC chromatograms. No signals were observed in the range of 2,000–10,000 Da, therefore, those mass spectra were not included in this article.

## Discussion

In this study, the antifungal activity of melanins from different biological origins was tested. To date, this is the first study to correlate the variation of the antifungal activity with the physicochemical characteristics of melanins.

It has been previously described that the efficiency of the melanin extraction from different biological origins is low due to factors associated with the molecule and the source (Ito and Wakamatsu, 2003). In the present work, a sample of human hair from a healthy volunteer was used as a source of human melanin. In this case, the melanin extraction efficiency reached to 3.6% (around 0.5 grams) which is similar to a previous study (Liu et al., 2003). However, the extraction efficiency of fungal melanin from cultures reached about 2% (5–10 mg) in the current study, which is low compared to another previous study (Helan et al., 2013).

In the current study, human and synthetic melanin showed an important inhibitory activity against all tested *Cryptococcus* strains (**Figure 2** and **Table 1**). In addition, this antifungal activity against the clinical isolates, obtained from patients, was higher than the reference strains (**Figure 3**). In previous work (Tapia et al., 2014), we demonstrated that melanin participates in a first line of the innate immune response of melanocytes stimulated with *Candida* extracts, by cell melanisation, which is related to the expression of Toll Like Receptor 4 (TLR4). Therefore, these results support the idea that human melanin can represent a mechanism of antimicrobial defense against fungi.

Similar to our preceding results obtained against *Candida* species (Fuentes et al., 2014), the average of MICs obtained with the synthetic melanin was slightly increased, with an equivalent range (6.25, 9.2, and 17.3 μg/mL for *C. albicans*, *C. neoformans*, and *C. gattii*, respectively). Although that *Candida* sp and *Cryptococcus* sp are two distinct microorganisms, this data suggests that *Candida* species would be more sensitive to synthetic melanin than *Cryptococcus* species. This might be explained by the structural differences between both yeasts at the fungal wall composition level and/or by the absence-presence of a capsule.

Similarly, to synthetic melanin, the MICs obtained with the human melanin were slightly lower in *C. neoformans* than *C. gattii* (13.7 versus 19.1 μg/mL in average, respectively), suggesting that not all strains of *Cryptococcus* show the same sensibility for melanin.

The antifungal activity of melanin extracted from *Cryptococcus* strains was significantly lower than those extracted from human and synthetic melanins. Human melanin showed an activity 14 times higher than the cryptococcal melanins. In addition to strain dependent effect, these results show that the antifungal activity of melanin is also dependent on the originating organism. In the current study, we tested the antifungal activity of fungal melanin extracts against the fungal species where they had been extracted from, showing that the activity was lower against strains of their own species. However, this doesn’t exclude, that they may have a better activity against other fungal species. This should be subject to further studies.

The physicochemical characterization of extracts revealed that melanin was the main component in all the analyzed extracts. The EDX analyses showed that the extracts were mainly made up of carbon and oxygen (65 and 35% on average, respectively) as well as the synthetic melanin, except for the presence of sodium (5%) in synthetic melanin (**Figure 4**). Although nitrogen is also present in the melanin structure, the proportion of N atoms in the melanin molecule is relatively low with respect to the other majority elements. The detection limit of EDX technique is 0.1% wt, and probably nitrogen content found in the sample was under this detection limit. In addition, the FITR spectrum of fungal melanin extracts were similar to synthetic melanin and in turn, similar to melanin analyzed in previous studies (**Figure 5**) (Morzyk-Ociepa, 2009; Helan et al., 2013; Banerjee et al., 2014; Sun et al., 2016). Similarly, the extracts analyzed by MALDI-TOF-MS presented similar spectra with synthetic melanin, particularly in the appearance of two ions of 656 and 681 Da (**Figure 7**). The chromatograms of the melanin extracts obtained by HPLC showed a profile with a single signal with the synthetic melanin (**Figure 6**). These results are consistent with previous studies (Sun et al., 2016). In effect the HPLC patterns of human and fungal melanins were similar to the synthetic melanin obtained through the oxidation of tyrosine, which means that both human and fungal melanins contain eumelanin as their principal element, with a similar structure to the melanin standard (synthetic melanin). Collectively these results obtained using several methods confirm that the herein studied extracts were formed of melanin.

Human and fungal melanin both present different antifungal activity and morphological differences, suggesting that antifungal activity may be correlated with the structure of the melanins. However, we could not associate the morphological differences between human and fungal melanin with their antifungal activity. Similarly, to previous studies (Liu et al., 2003; Costa et al., 2012), the human melanin was compact maintaining its characteristic ellipsoidal shape of synthetizing vesicle (melanosome). This structural form may increase the antifungal activity to the melanin in contrast to the form of fungal melanin extracts. However, the structural form of the fungal melanins was similar to the synthetic melanin, which showed a similar antifungal activity as the human melanin. In this context, it is difficult to link the amorphous or compact disposition of the human melanin with its antifungal activity. Thus the differential antifungal activities between melanins from different sources might be due to chemical characteristics.

The HPLC and FTIR analysis showed that the melanin extracted from *Cryptococcus* species displayed physicochemical differences and similarities in comparison with the synthetic or human melanin. In the chromatograms obtained by HPLC, a mains and single peak was observed for the synthetic melanin with a retention time (Rt) of 9.606 min. Human melanin extracts also showed a main peak at Rt = 9.481 min by HPLC analysis, suggesting that human melanin extract contains an abundant compound related closely to melanin (**Table 3** and **Figure 6**). On the other hand, fungal melanin extracts showed also a main compound eluted in a range from 9.3 to 12.0 min and a minor chromatographic peak eluted approximately at 13 min. It suggested that the different fungal melanin extracts could contain more than one melanin species or derivatives. Some of them are related closely to the synthetic melanin and some of them differ slightly in the retention time in comparison with synthetic melanin. This is supported by the analysis through MALDI-TOF-MS, due to the presence of an abundant ion (656 Da), representing the main compound in the samples, followed by a less intense ion (861 Da) and a differential very low intense ion (825 Da), which was not observed in all mass spectrum (**Figure 7**). However, the presence of this low intense ion was not associated with a higher or lower antifungal activity.

Fourier transform infrared spectroscopy analysis also revealed that fungal melanin presents differences with respect to human melanin in the zone of the aromatic ring. The intensity of the vibrations modes corresponding to C=C and C=N bonds of the aromatic ring appear decreased in the fungal melanins that may be explained by the lower melanin concentration in the fungal sample, the quality of the purification (presence of impurities) or the interactions of impurities with the aromatic bonds. Interestingly the melanin spectrum of *C. gattii* VGIII WM175 presented two marked peaks in the FTIR aromatic region (**Figure 5C**), which are scarcely present in the rest of the fungal melanins. Similarly, this melanin from *C. gattii* VGIII WM175 showed a higher antifungal activity than the melanin obtained from the other tested cryptococcal molecular types/species. The physicochemical difference observed by FTIR technique specifically for melanin extracted from *C. gattii* VGIII WM175 could explain it differential antifungal activity. Other studies of FTIR in melanin have obtained more detailed spectra with signals in 3385, 3205, 2910, 2362, 1714, 1622, 1396, and 1295 cm^-1^ (Alaluf et al., 2002; Helan et al., 2013). The differences obtained through previous studies could be related to the origin of the melanin obtained, the initial substrate used for the melanization, the methods of extraction and purification and even the FTIR equipment utilized. It was reported that the acid/alkali method that we have used in our study, could affect the structure of the human melanin compared to enzymatic method. Although the usual enzymatic method was reported to be more efficient (Liu et al., 2003) since higher concentration can be extracted, we obtained similar efficiently with the acid/alkali method that is less costly and we did not observe any impair in the melanin morphology regarding the data of the SEM. Nevertheless, this discrepancy with other studies supports that structural differences exist between the melanins synthetized by different organisms that can affect their function. The MALDI-TOF MS technique revealed the presence of two ions at 825 Da (**Figure 7**) for some melanins, but we could not associate this chemical difference with a higher or lower antifungal activity. However, the analysis of more fungal melanins may allow observing a correlation.

## Conclusion

In summary, we confirm the potential protective role of human melanin, especially in the innate immunity against fungi and its differential activity compared to cryptococcal melanins. Melanins extracted from the human pathogenic *Cryptococcus* species showed a lower fungal activity against their own genus. Although some physical-chemical differences were found, they do not explain the differences in the antifungal activity against *Cryptococcus* of human and cryptococcal melanins. More detailed studies on the structure should be considered to associate structure and antifungal activity. Herein presented results open a new therapeutic approach against fungal infections but more researchs are need to elucidate the antifungal mechanism of melanin.

## Author Contributions

NC: Work design, melanin extraction, anti-fungal susceptibility testing, physical-chemical characterization experiments and work writing. CC: physical-chemical experiments management (SEM, EDX, and FITR). PR: work design and critical review of the manuscript. GH: work design and critical review of the manuscript. VO: physical-chemical characterization through MALDI-TOF-MS experiments performance and critical review of the manuscript. CV: physical-chemical characterization through HPLC experiments performance and critical review of the manuscript. WM: work design, strains donation and critical review of the manuscript. FM: work design and critical review of the manuscript. CT: work design, theses director, melanin extraction, anti-fungal susceptibility testing direction and work writing.

## Conflict of Interest Statement

The authors declare that the research was conducted in the absence of any commercial or financial relationships that could be construed as a potential conflict of interest.
